# Intelligence Polygenic Score Is More Predictive of Crystallized Measures: Evidence From the Adolescent Brain Cognitive Development (ABCD) Study

**DOI:** 10.1177/09567976231160702

**Published:** 2023-05-05

**Authors:** Robert J. Loughnan, Clare E. Palmer, Wesley K. Thompson, Anders M. Dale, Terry L. Jernigan, Chun Chieh Fan

**Affiliations:** 1 Department of Cognitive Science, University of California San Diego; 2 Population Neuroscience and Genetics, University of California San Diego; 3 Center for Human Development, University of California San Diego; 4 Center for Population Neuroscience and Genetics, Laureate Institute for Brain Research; 5 Center for Multimodal Imaging and Genetics, University of California San Diego School of Medicine; 6 Department of Radiology, University of California San Diego School of Medicine; 7 Department of Neuroscience, University of California San Diego School of Medicine

**Keywords:** behavior genetics, childhood development, cognitive ability, open materials

## Abstract

Findings in adults have shown that crystallized measures of intelligence, which are more culturally sensitive than fluid intelligence measures, have greater heritability; however, these results have not been found in children. The present study used data from 8,518 participants between 9 and 11 years old from the Adolescent Brain Cognitive Development (ABCD) Study. We found that polygenic predictors of intelligence test performance (based on genome-wide association meta-analyses of data from 269,867 individuals) and of educational attainment (based on data from 1.1 million individuals) predicted neurocognitive performance. We found that crystallized measures were more strongly associated with both polygenic predictors than were fluid measures. This mirrored heritability differences reported previously in adults and suggests similar associations in children. This may be consistent with a prominent role of gene–environment correlation in cognitive development measured by crystallized intelligence tests. Environmental and experiential mediators may represent malleable targets for improving cognitive outcomes.

Scores on cognitive tests in both children and adults have been linked to long-term outcomes and to genetic variation (Deary & Gottfredson, 2004; Kell et al., 2013; Kuh et al., 2004; Makel et al., 2016; Polderman et al., 2015). Some cognitive tests, such as those requiring literacy and mathematical skills, depend on and are more sensitive to variability in cultural and socioeconomic factors. These measures are often referred to as *crystallized* intelligence measures. In contrast, other tests that tap the capacity to solve novel problems or process novel information, often referred to as *fluid* measures, are less culturally sensitive and are less strongly related to socioeconomic variables (Akshoomoff et al., 2013, 2014). A recent review reported systematic differences in heritability (an estimate of trait variability attributable to genetic variation) of the traits measured by these different kinds of cognitive measures (Kan et al., 2013). Surprisingly, in studies of adult twins, more culturally sensitive tests exhibited higher heritability, which runs counter to predictions from conventional models of intelligence. The authors described similar trends in the twin studies of children, but increased heritability of crystallized relative to fluid measures has not yet been established for children, in whom intellectual functions are continuing to mature.

The finding that the measures most strongly influenced by cultural factors exhibit higher heritability is perhaps counterintuitive; however, previous authors have noted that genetic variation can be associated with environmental, cultural, or experiential factors that themselves amplify effects of a genotype on the phenotype, a phenomenon often referred to as *gene–environment correlation*. These associations between genotypes and environmental, cultural, or experiential factors could influence the development of cognitive and intellectual abilities in several ways. As an example, if other people in the social environments of children recognize traits (e.g., precocious behavior) in those with a genetic propensity for a given cognitive ability, they may begin to treat such individuals differently, rewarding them disproportionately for intellectual pursuits, investing more in their instruction, and/or placing them in environments that drive learning more effectively. Alternatively, the associations can be driven by the motivation of the children themselves if, for example, they develop greater enthusiasm for intellectual activities for which they have been more frequently rewarded and that they then pursue more assiduously, thus enjoying beneficial effects of the increased practice associated with these activities. In either case, the genetically advantaged abilities are disproportionately enhanced by these mediating environmental, cultural, or experiential factors. Of course, individuals with less advantageous genotypes may experience the converse of these social and motivational effects, resulting in languishing, or in the worst case suppressed, intellectual development, even within similar environments. Such gene–environment correlation effects can increase variance in intellectual phenotypes and increase estimates of heritability using both epidemiological and genomic methods (Beam & Turkheimer, 2013). The important implication is that a component of this increased heritability requires the mediating environmental, cultural, or experiential effects for its expression. In essence, more direct biological effects of the genotype *and* associated differences in the environments or experiences of the child are both contributing causal factors influencing the mature phenotype, but they act through dissociable mechanisms.

**Statement of Relevance**

Genetics are known to have a moderate influence on intelligence test performance. Two types of intelligence that are often distinguished are (a) crystallized intelligence, which is related to acquired knowledge and is thought to be more linked to culture (e.g., reading ability), and (b) fluid intelligence, which is related to problem solving in unfamiliar situations and is thought to be less linked to culture (e.g., solving a puzzle). Conventional theories predict that crystallized measures of intelligence, which are more culturally sensitive, should be more determined by one’s environment and therefore should have a smaller genetic contribution than fluid measures, which are less culturally sensitive. The work reported in this article provides evidence in children that the inverse may be true. One possible explanation for this surprising finding is that crystallized measures of intelligence exhibit a greater degree of gene–environment correlation. This can be illustrated by a very simple example: Perhaps society more readily provides avenues for strong readers to read than strong puzzle solvers to solve puzzles. Such a mechanism would increase the amount we estimate that genetics contribute to culturally sensitive crystallized measures. If this does explain these findings, then this may provide potential avenues of environmental and/or experiential modifiable factors that affect the relationship between genetics and intelligence.

Heritability is a population statistic frequently measured using a twin design. For this study, we used polygenic scores to examine variation in genetic and experiential factors and their relationship to trait measures of cognitive function. The advantage of polygenic scores is that they can be used to index relevant genetic factors in samples of unrelated individuals by leveraging the statistical power of meta-analysis results from large genome-wide association studies (GWASs). The present research used demographic information, neurocognitive test scores, and genomic data from a large sample of 8,518 children between 9 and 11 years old from the Adolescent Brain Cognitive Development (ABCD) Study. We examined polygenic scores of intelligence test performance (based on GWASs of 269,867 individuals; Savage et al., 2018) and educational attainment, sometimes considered a proxy for intellectual ability (based on 1.1 million individuals; Lee et al., 2018), to address the following question: Do these genomic predictors account for more of the variability in estimates of culturally sensitive crystallized traits than fluid traits in children, as might be expected from reports of higher heritability in adult twins?

In additional analyses, we examined the degree to which the findings in the ethnically diverse ABCD sample were similar between the subgroup of children with high genomic European ancestry and the remaining subgroup of children who were from diverse ancestry groups. Finally, using simulations, we tested whether our observed findings may be due to previously reported differences in test–retest reliabilities (for crystallized vs. fluid measures).

## Open Practices Statement

The ABCD data set can be accessed by approved researchers at https://nda.nih.gov/abcd. A Jupyter Notebook of the analysis can be found at https://github.com/robloughnan/ABCD_Intelligence_Polygenic_Score. The design and analysis plan for this study were not preregistered.

## Method

### Data available in ABCD Data Release 2.0.1

The ABCD study (http://abcdstudy.org) enrolled the families of 11,875 children between 9 and 10 years old at baseline (Morgan et al., 2017). This longitudinal study follows the development of these children at 21 sites across the United States for 10 years. The cohort exhibits a large degree of sociodemographic diversity. Exclusion criteria were limited to (a) lack of English proficiency; (b) the presence of severe sensory, neurological, medical, or intellectual limitations that would inhibit the child’s ability to comply with the protocol; and (c) an inability to complete an MRI scan at baseline. The study protocols are approved by the University of California San Diego Institutional Review Board (Auchter et al., 2018). Parent/caregiver permission and assent from each child participant were obtained. Here, our data were drawn from the baseline assessments shared in ABCD Data Release 2.0.1 (https://doi.org/10.15154/1504041). The University of California San Diego Institutional Review Board stated that analysis of ABCD data does not constitute human subjects research, as data have been deidentified, and studies of these data have therefore been deemed exempt from review.

#### Cognitive measures

Seven of the 10 cognitive tasks were subtests from The NIH Toolbox Cognition Battery (NTCB) in the version recommended for ages 7+ (http://www.nihtoolbox.org; Weintraub et al., 2013). The average time to complete this battery is approximately 35 min. The NTCB was administered in English (Casaletto et al., 2015) using an iPad, with support from a research assistant when needed. The battery yields individual test scores measuring specific constructs and composite scores that have been shown to be highly correlated with gold-standard measures of intelligence in adults (Heaton et al., 2014) and children (Akshoomoff et al., 2013). Here, all seven individual test scores and two composite scores were examined: the *crystallized cognition* composite score (derived from scores on the Picture Vocabulary and Oral Reading Recognition measures) and the *fluid cognition* composite score (derived from the remaining measures). Additionally, three neurocognitive tasks were used that were not components of the NTCB: Rey Auditory Verbal Learning Test, little-man task, and Matrix Reasoning. See the Supplemental Material available online for a description of each task.

#### Latent neurocognitive factors

Thompson et al. (2018) derived an orthogonal three-factor, varimax-rotated solution for the latent structure across the neurocognitive battery in ABCD using Bayesian probabilistic principal components analysis. The latent factor solution included nine of the 10 measures described above, excluding the Matrix Reasoning task, which had very little effect on the solution. Figure S1 in the Supplemental Material shows factor loadings across included measures. These factors will be referred to as Bayes Factors (BFs) 1 to 3. Language tasks loaded most heavily on BF 1, which was highly correlated with the crystallized composite (*r* = .93); executive functioning tasks loaded most heavily on BF 2; and learning/memory tasks loaded heavily on BF 3. BFs 1 to 3 respectively explained 21.1%, 20.4%, and 18% of the variance in included measures.

#### Genetic data and computing polygenic scores

Using genotype data, we derived genetic ancestry using *fastStructure* (Raj et al., 2014) with four ancestry groups. Genetic principal components were also calculated using *PLINK* for use as covariates in statistical models (see the Supplemental Material). Variants were imputed using the Michigan Imputation Server (Das et al., 2016). Polygenic scores were computed using *PRSice* (Euesden et al., 2015). The intelligence polygenic score (IPS) was trained on 269,867 individuals by Savage et al. (2018) and focused on neurocognitive tests considered to gauge fluid intelligence. The educational attainment polygenic score (EAPS) was generated from 1.1 million individuals, predicting the phenotype of number of years of schooling completed. See the Supplemental Material for further details on genetic data and analysis.

We were primarily focused on studying the IPS association with cognitive tests in ABCD because it was trained on a more directly relevant phenotype. However, we examined EAPS in a secondary analysis for comparison because it has previously been used as a proxy for cognitive ability and has a discovery sample size 4 times that of the IPS.

### Analytic methods

#### Ancestry group analyses

Training and testing polygenic scores in different ancestry groups has been shown to reduce predictive power (Carlson et al., 2013; Duncan et al., 2019; Martin et al., 2017). Given the ancestry differences between the polygenic score discovery samples (predominantly European) and the ABCD Study (multiple ancestry groups), we wanted to confirm that our main results in the full samples were not driven by population structure. Thus, we additionally performed analyses in two subsamples: (a) children with an estimated proportion of European ancestry higher than 90% (European ancestry) and (b) a group of the remaining children with diverse ancestry, which included those from other or mixed ancestry (diverse ancestry).

#### Statistical model for genomic prediction of behavioral measures

To assess the association between the polygenic scores and cognitive performance in ABCD, we fitted generalized linear mixed-effects models using the *gamm4* package (Wood & Scheipl, 2014) in R. Each model predicted performance on a different cognitive measure and factor score (for BFs 1–3). Continuous variables were *z*-scored before model fitting to allow coefficients to be interpreted as standardized effect sizes. To test whether regression coefficients differed between regressions, we performed a *z* test on the difference between coefficients, based on the propagated standard error for the two regression coefficients as the sum of the error of variances for each measure. This test assumes that standard errors are uncorrelated and so provides a conservative estimate of significance. See the Supplemental Material for details and covariates used.

## Results

### Demographics

**Fig. 1.**
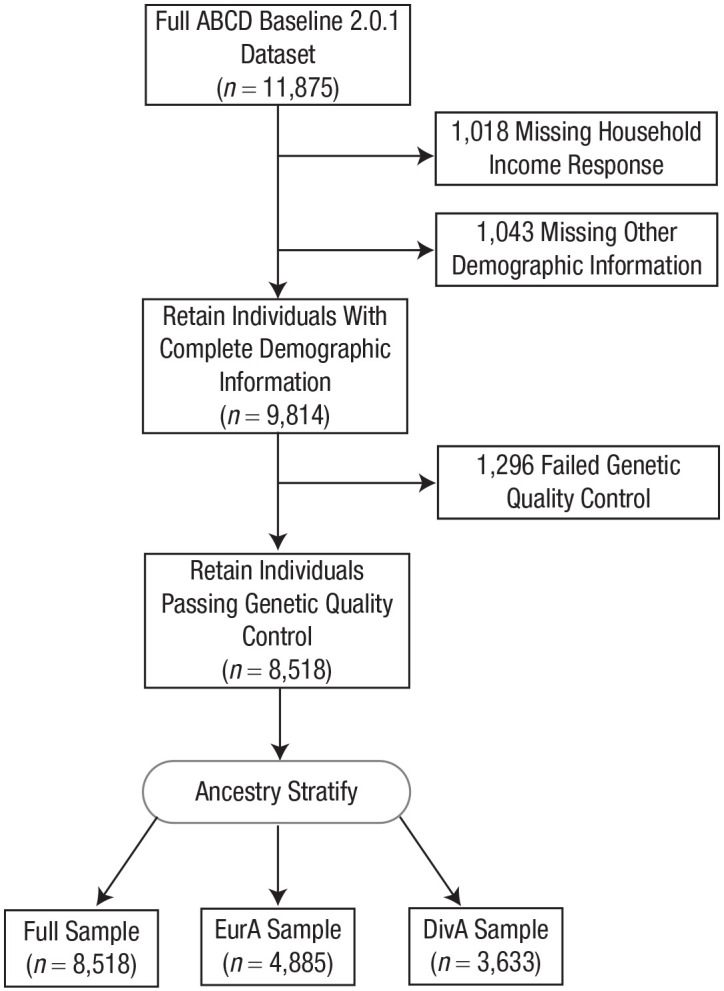
Flowchart showing procedures for sample selection and exclusion. ABCD = Adolescent Brain Cognitive Development; EurA = European ancestry; DivA = diverse ancestry.

Figure 1 shows a flowchart for sample selection. For the final analysis, we had 8,518 individuals in the full sample, 4,885 in the European ancestry sample, and 3,633 in the diverse ancestry sample. Table 1 shows demographic statistics for the full sample and for each subsample.

**Table 1.** Demographic Statistics for the Full Sample for the Present Genomic Prediction Analyses and for the Genomic European Ancestry and Genomic Diverse Ancestry Subgroups

Variable	Full sample (*N* = 8,518)	European ancestry subgroup (*n* = 4,885)	Diverse ancestry subgroup (*n* = 3,633)
Age in months, *M* (*SD*)	119.05 (7.48)	119.21 (7.49)	118.85 (7.47)
Sex male, *n* (%)	4,438 (52.1)	2,576 (52.7)	1,862 (51.3)
Married parents, *n* (%)	6,024 (70.7)	4,066 (83.2)	1,958 (53.9)
Parental education, *n* (%)			
< High school diploma	302 (3.5)	21 (0.4)	281 (7.7)
High school diploma/GED	649 (7.6)	138 (2.8)	511 (14.1)
Some college	2,149 (25.2)	899 (18.4)	1,250 (34.4)
Bachelor’s degree	2,318 (27.2)	1,548 (31.7)	770 (21.2)
Postgraduate degree	3,100 (36.4)	2,279 (46.7)	821 (22.6)
Annual household income, *n* (%)			
< $50,000	2,353 (27.6)	596 (12.2)	1,757 (48.4)
≥ $50,000–$99,999	2,444 (28.7)	1,471 (30.1)	973 (26.8)
≥ $100,000	3,721 (43.7)	2,818 (57.7)	903 (24.9)
Race, *n* (%)			
White	5,715 (67.7)	4,750 (97.4)	965 (27.1)
Black	1,129 (13.4)	1 (0.0)	1,128 (31.7)
Asian	199 (2.4)	0 (0.0)	199 (5.6)
Other	1,397 (16.6)	128 (2.6)	1,269 (35.6)
Hispanic, *n* (%)	1,628 (19.1)	131 (2.7)	1,497 (41.2)

### Behavioral measures and sociocultural factors

**Fig. 2.**
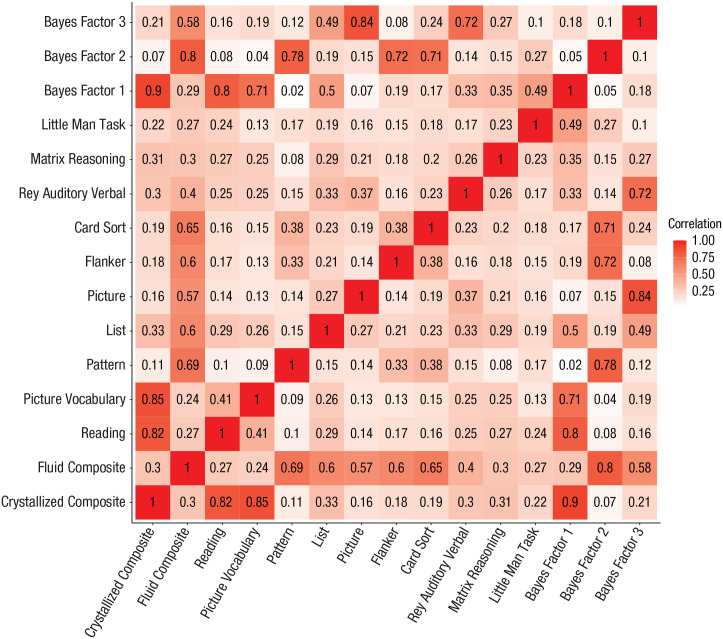
Partial correlation matrix showing intercorrelations among individual task performance measures (controlling for covariates included in subsequent analyses: age, sex, parents’ marital status, parental education, household income, principal components of genetic ancestry, and Hispanic status) in the full sample included in the present study.

Mean performance, standard deviation, median, and estimates of variance explained by age, sex, and the set of sociocultural covariates (parents’ marital status, highest education level of parent/caregiver, household income, ethnicity, genetic principal components) are given for each behavioral measure examined in Table 2. Consistent with previous reports, results showed substantial differences in the degree to which sociocultural factors account for variability in these measures. The crystallized composite, its constituent Picture Vocabulary and Reading Recognition measures, and BF 1, on which these measures of language and literacy load heavily, all exhibited higher levels of association with sociocultural variables. This pattern persisted when analyses controlled for IPS (Table S2 in the Supplemental Material). Partial correlations between the individual cognitive task measures in analyses controlling for covariates (Fig. 2) suggest that performance on the different tasks is modestly correlated across children (*r*s = .08–.41) in this sample. Correlations peaked in the .3 range within fluid composite measures, and the highest correlation was observed between the two crystallized composite measures (Picture Vocabulary and Oral Reading Recognition: *r* = .41).

**Table 2.** Descriptive Statistics for Each Behavioral Measure and Estimated Percentage of Variance Explained by Sex, Age, and the Set of Sociocultural Covariates (Parents’ Marital Status, Parental Education, Household Income, Genetic Ancestry Principal Components, and Hispanic/Non-Hispanic) in the Full Sample

Measure	*M* (*SD*)	*Mdn*	Percentage of variance explained		
			Sex	Age	Sociocultural covariates
Crystallized composite	86.87 (6.93)	87	0.01	9.51	21.57
Fluid composite	92.18 (10.43)	93	0.32	7.17	10.28
Reading	91.23 (6.73)	91	0.01	5.97	13.18
Picture Vocabulary	85.04 (8.02)	84	0.07	7.67	20.14
Pattern	88.29 (14.47)	88	0.57	4.81	1.90
List	97.43 (11.81)	97	0.13	2.04	9.47
Picture	103.33 (12.01)	103	0.51	1.17	5.46
Flanker	94.42 (8.83)	96	0.03	3.21	3.73
Card Sort	92.97 (9.26)	94	0.48	3.76	5.22
Rey Auditory Verbal Learning	43.78 (9.96)	44	1.25	2.23	8.57
Matrix Reasoning	18.13 (3.74)	18	0.34	2.74	9.15
Little-man task	0.60 (0.17)	0.56	0.48	5.13	6.25
Bayes Factor 1	0.05 (0.76)	0.06	0.28	9.63	20.85
Bayes Factor 2	0.02 (0.76)	0.06	0.22	5.49	2.65
Bayes Factor 3	0.04 (0.70)	0.04	0.89	1.59	7.83

### Genomic prediction of crystallized and fluid cognition measures

Table 3 summarizes the results of regressions predicting the crystallized and fluid composites with IPS or EAPS in the full sample, separately for the European ancestry and diverse ancestry subsamples. The IPS was a significant predictor of both measures in all analyses. Importantly, the standardized regression coefficient was significantly higher for the crystallized than the fluid composite regardless of ancestry group (full sample: *z* = 4.8, *p* = 1.8 × 10^–6^, European ancestry: *z* = 4.6, *p* = 5.1 × 10^–6^, diverse ancestry: *z* = 2.5, *p* = 1.4 × 10^–2^).

**Table 3.** Regression Results for Generalized Linear Mixed-Effects Models Associating Intelligence Polygenic Score and Educational Attainment Polygenic Score With Crystallized Composite and Fluid Composite of the NIH Toolbox Cognition Battery in the Full Sample and Ancestry Subgroups

Sample	Fluid composite				Crystallized composite			
	β	*t*	*p*	Variance explained (%)	β	*t*	*p*	Variance explained (%)
Intelligence polygenic score								
Full sample	0.28	8.03	1.14 × 10^–15^	0.75	0.50	15.82	1.31 × 10^–55^	2.86
European ancestry subgroup	0.11	7.53	6.10 × 10^–14^	1.15	0.21	14.48	1.44 × 10^–46^	4.13
Diverse ancestry subgroup	0.20	3.41	6.52 × 10^–4^	0.32	0.40	7.34	2.68 × 10^–13^	1.47
Educational attainment polygenic score								
Full sample	0.11	7.23	5.26 × 10^–13^	0.61	0.19	14.21	2.56 × 10^–45^	2.32
European ancestry subgroup	0.09	6.60	4.66 × 10^–11^	0.89	0.18	12.95	9.36 × 10^–38^	3.34
Diverse ancestry subgroup	0.08	3.38	7.28 × 10^–4^	0.32	0.15	6.66	3.24 × 10^–11^	1.21

In no case did EAPS, despite a much larger training sample size, appear to account for more of the variance in the neurocognitive measures than did IPS. However, across ancestry groups and for both composite scores, combining both genomic predictors explained significantly more variance in behavior than IPS alone (see the Supplemental Material). IPS and EAPS combined explained 5.8% of the variance (*p* = 4.5 × 10^–64^) in the crystallized composite for European ancestry (a 40% increase compared with IPS alone). Figure S2 and Tables S3 through S8 in the Supplemental Material show regression results for each behavior using IPS, EAPS, and IPS and EAPS combined within each ancestry group.

**Fig. 3.**
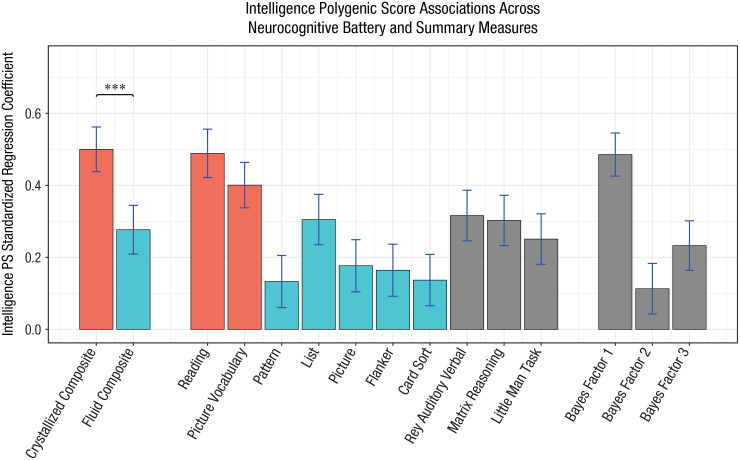
Standardized regression coefficients of intelligence polygenic score (IPS) fitting linear mixed models to performance on fluid and crystallized composites, each individual task from the NIH Toolbox Cognition Battery, additional measures from the Adolescent Brain Cognitive Development (ABCD) Study neurocognitive battery, and Bayes (latent) Factors 1 to 3 in the full sample. Prediction of the crystallized composite is significantly stronger than for the fluid composite (***p < .001). Tasks included in the fluid composite (shaded in blue) have consistently lower regression coefficients than those included in the crystallized composite (shaded in red). Additional measures from the neurocognitive battery exhibit associations with IPS more similar to the fluid composite than to the crystallized composite; however, Bayes Factor 1, on which the verbal tasks load heavily, exhibits an association similar to the crystallized composite. Error bars show estimates of 95% confidence intervals as 1.96 × standard error.

Fitting separate regression models for each individual task in the neurocognitive battery, we found that IPS was a significant predictor for each cognitive measure for the full sample and the European ancestry subsample (all *p*s < 10^–3^), surviving the Bonferroni-corrected significance threshold of .05/10 = .005. Within the diverse ancestry subsample, performance on only six of the 10 tasks was individually significantly predicted by IPS (Table S8 in the Supplemental Material). Figure 3 shows the standardized regression coefficients of IPS predicting performance on each task as well as the crystallized and fluid composite measures from the NTCB and BFs 1 to 3 (Thompson et al., 2018) in the full sample. Individual cognitive measures included in the crystallized composite have consistently higher IPS standardized regression weights than the measures included in the fluid composite. Other neurocognitive tasks from the ABCD battery (shaded in gray) showed similar associations to the fluid composite. The results for the BFs mirrored these results: BF 1, on which crystallized measures had the highest factor loadings (Figure S1; Thompson et al., 2018), displayed a stronger association with IPS than BF 2 and BF 3, on which fluid, executive function, and memory measures had higher loadings. The results in the European ancestry and diverse ancestry subsamples are provided in the Supplemental Material.

### Sensitivity analyses to address test reliability

A previous study found that test–retest reliability for the fluid composite from the NTCB (.76) was somewhat lower than for the crystallized composite (.85; Akshoomoff et al., 2013). This raises questions about whether differences in the strength of the composites’ associations with IPS could be attributed to more noise in the fluid composite measure. In supplementary sensitivity analyses, we demonstrated that our results are robust to the addition of simulated noise to the crystallized composite that mimics this difference in test reliability. At this level of simulated noise, we estimated 100% power (α = .05) to detect a stronger IPS association with crystallized performance than with fluid performance. This analysis is described in detail in the Supplemental Material.

## Discussion

We showed that polygenic predictors of intelligence test performance and of educational attainment are associated with neurocognitive performance in this large group of children from diverse backgrounds. These results are consistent with previous findings demonstrating that virtually all behavioral traits, including cognitive and intellectual phenotypes, have substantial genetic components (Turkheimer, 2000). Given that behavioral phenotypes emerge through interactions between children and their physical, social, and cultural environments, much attention has been paid to how these environmental factors modify the phenotypes, given that they are presumably the malleable factors. However, recently, more attention has focused on the possible roles of mediating nongenetic (environmental, cultural, or experiential) factors that, through their statistical association with genetic variation (gene–environment correlation), may amplify heritability (Beam & Turkheimer, 2013; Kan et al., 2013).

We found that a culturally dependent estimate of crystallized cognitive functions, the crystallized composite measure from the NTCB, was more strongly associated with the best available polygenic predictor of intelligence test performance than the fluid composite measure was, consistent with earlier findings in adults of heritability differences (Kan et al., 2013) and polygenic score performance (Genç et al., 2021) across similar measures. This is despite the IPS being based on a large meta-analysis of GWASs combining cognitive measures that were described by the authors as primarily fluid intelligence measures (Savage et al., 2018). Indeed, the relative size of the IPS association across the 15 measures examined here (Fig. 2) closely mirrored the relative percentage of variance explained in these measures by sociocultural variables (Table 2), a pattern that persisted after we accounted for IPS (Table S2). These results may be consistent with previous descriptions of gene–environment correlation effects and with analyses by Beam and Turkheimer (2013), in which these more sociocultural measures of intelligence exhibit a greater degree of gene–environment correlation, thereby amplifying the degree to which we estimate their genetic contribution. These authors also showed that increasing gene–environment correlation over time could explain observed increases in the heritability of measures of cognitive function through development. The ABCD Study will provide an opportunity to measure changes in heritability at later time points of this longitudinal study. Importantly, despite the lower test–retest reliability of the fluid compared with the crystallized composite score from the NTCB (Akshoomoff et al., 2013), our supplementary analyses show that this difference in test reliability is unlikely to explain our findings. Nevertheless, across different social and cultural contexts, we may expect to observe variable reliability estimates for fluid and crystallized measures. This could, in turn, affect the degree to which fluid and crystallized measures have a differential genetic loading. Once again, future time points of the ABCD Study will enable us to quantify the reliability of these measures and the degree to which this could explain our findings.

We also showed that generally across neurocognitive measures, the IPS had higher predictive performance than the EAPS (Tables S3–S8). One may have predicted that EAPS would have been a more powerful predictor of cognitive measures in ABCD than IPS because it had more than 4 times the discovery sample size. We interpret our finding of the IPS generally having stronger associations as being due to the phenotype being a better match between training and testing—rather than being a proxy measure. This is in agreement with a recent study in young adults in Germany that found IPS to be more predictive of cognitive performance than EAPS (Genç et al., 2021), but it contrasts with results in a similar analysis in children and adolescents from the United Kingdom (Allegrini et al., 2019). This discrepancy may be due to methodological differences; alternatively, sociodemographic differences between countries may be the key factor explaining these inconstancies. Educational attainment, although clearly related to scores on cognitive tests, may be influenced by other genetically influenced traits (e.g., personality) that may contribute to greater persistence in formal education; thus, EAPS is likely to reflect these traits to a greater degree. Such pleiotropic EAPS effects have been observed in adults (Krapohl et al., 2014). When we included both EAPS and IPS in a single model, they together explained 5.8% of the variability in the crystallized composite (European ancestry; Table S6 in the Supplemental Material), substantially more than IPS alone explained (4.1%), indicating that these genomic predictors capture unique sources of the relevant variance and are likely measuring somewhat different (relevant) constructs.

The possible role of gene–environment correlation in our finding of crystallized measures being more strongly predicted by genomic predictors than fluid measures is deserving of further investigation. Identifying environmental, cultural, or experiential factors that contribute to heritability of cognitive and intellectual phenotypes is important because it can point to practices that better adapt to neurogenetic diversity among children. Innovative pedagogical practices may lead to approaches that increase “enhancing” environmental, cultural, or experiential effects in the subset of children disadvantaged by current practices and reduce environmental, cultural, or experiential effects that suppress intellectual development and academic achievement, which may lead to more equitable educational outcomes.

## Limitations and Caveats

The proportion of variance in the cognitive measures accounted for by the genomic predictors was larger in the European ancestry participants than in the diverse ancestry group (Tables S5 and S7 in the Supplemental Material), as would be expected given that the discovery samples were in individuals of European ancestry. However, the patterns were generally similar in the diverse ancestry group. This suggests similar genetic architecture for these cognitive phenotypes across ancestry groups and supports the validity of the results from the full sample. In all three groups, analyses included the top 10 genetic principal components derived from the full sample as covariates. Because of broad ancestral diversity in the ABCD cohort, there was limited power for comparing the effects in different ancestry groups. As has been discussed in genetics generally (Martin et al., 2019; Petrovski & Goldstein, 2016), the lower predictive performance in the diverse ancestry group once again underscores the importance of collecting genetic data from ancestrally diverse populations and developing methods that can be used across ancestry groups.

Recent work appears to show that a sizable proportion of the association between EAPS/IPS and cognitive outcomes may manifest through indirect effects (assortative mating, population stratification, and environmentally mediated parental genetic effects) versus direct effects (inherited genetic variation; Howe et al., 2022; Okbay et al., 2022; Selzam et al., 2019). We attempted to mitigate this confound by controlling for socioeconomic variables. However, in addition to the described possible role of gene–environment correlation explaining the differential association of IPS with crystallized versus fluid measures, it may be that these other indirect effects also to some degree inflated the association we found between EAPS/IPS and cognitive performance.

These results are consistent with previous evidence for a role of genetic variation in developing cognitive functions. However, it should be emphasized that the genomic predictors (together) accounted for only 4.15% of cognitive performance variance in the full sample. Furthermore, this was observed for the crystallized composite measure, the culturally sensitive measure hypothesized to exhibit increased genetic association as a result of gene–environment correlation effects. The additive effects of potentially confounding sociocultural covariates, even in analyses controlling for IPS, accounted for 13.2% of the variability. For the fluid composite, the genomic predictors together accounted for only 1.1% of the variance, and sociocultural covariates accounted for almost 5%. Of note, even with the narrow 2-year age range in the cohort, age alone accounted for 10% of the variability in the crystallized composite measure and 7% in the fluid composite measure. These effects may reveal clues about a highly dynamic process of cognitive and intellectual development in children.

## Supplemental Material

sj-docx-1-pss-10.1177_09567976231160702 – Supplemental material for Intelligence Polygenic Score Is More Predictive of Crystallized Measures: Evidence From the Adolescent Brain Cognitive Development (ABCD) Study Click here for additional data file. Supplemental material, sj-docx-1-pss-10.1177_09567976231160702 for Intelligence Polygenic Score Is More Predictive of Crystallized Measures: Evidence From the Adolescent Brain Cognitive Development (ABCD) Study by Robert J. Loughnan, Clare E. Palmer, Wesley K. Thompson, Anders M. Dale, Terry L. Jernigan and Chun Chieh Fan in Psychological Science

## Transparency

*Action Editor:* Karen Rodrigue

*Editor:* Patricia J. Bauer

*Author Contributions*

**Robert J. Loughnan:** Conceptualization; Data curation; Formal analysis; Investigation; Methodology; Software; Visualization; Writing – original draft; Writing – review & editing.

**Clare E. Palmer:** Methodology; Writing – review & editing.

**Wesley K. Thompson:** Methodology; Supervision; Writing – review & editing.

**Anders M. Dale:** Methodology; Supervision.

**Terry L. Jernigan:** Investigation; Methodology; Project administration; Supervision; Writing – original draft; Writing – review & editing.

**Chun Chieh Fan:** Funding acquisition; Methodology; Project administration; Resources; Supervision; Writing – review & editing.
